# Supplementary material for: Cannabis for the Treatment of Fibromyalgia: A Systematic Review
Source: Biomedicines. 2023 Jun 2;11(6):1621. doi: 10.3390/biomedicines11061621 (PMC10295750; doi:10.3390/biomedicines11061621)
Supplement: Supplementary file 1 [file biomedicines-11-01621-s001.zip › Table S1.pdf]

Table S1. Database Search strategy

Search strategies	Results
**MEDLINE**<br>**#1**: *"fibromyalgia"* OR *"central sensitization"* OR "chronic *pain"*<br>**#2**: *"medical cannabis"* OR *"cannabis"* OR *"cannabinoids"*<br>**#3**: *"randomized controlled trial"* OR *"controlled clinical trial"* OR "randomized" OR "placebo" OR "randomly" OR "trial"<br>**#4**: #1 AND #2 AND #3	363
**EMBASE**<br>**#1**: *"fibromyalgia"* OR *"central sensitization"* OR *"chronic pain"*<br>**#2**: *"medical cannabis"* OR *"cannabis"* OR *"cannabinoids"*<br>**#3**: *"randomized controlled trial"* OR *"controlled clinical trial"* OR "randomized" OR "placebo" OR "randomly" OR "trial"<br>**#4**: #1 AND #2 AND #3	604
**Cochrane Central Register of Controlled Trials**<br>**#1**: *"fibromyalgia"* OR *"central sensitization"* OR *"chronic pain"*<br>**#2**: *"medical cannabis"* OR *"cannabis"* OR *"cannabinoids"*<br>**#3**: *"randomized controlled trial"* OR *"controlled clinical trial"* OR "randomized" OR "placebo" OR "randomly" OR "trial"<br>**#4**: #1 AND #2 AND #3	182
**Cochrane Database of Systematic Reviews**<br>**#1**: *"fibromyalgia"* OR *"central sensitization"* OR *"chronic pain"*<br>**#2**: *"medical cannabis"* OR *"cannabis"* OR *"cannabinoids"*<br>**#3**: *"randomized controlled trial"* OR *"controlled clinical trial"* OR "randomized" OR "placebo" OR "randomly" OR "trial"<br>**#4**: #1 AND #2 AND #3	21
**Scopus**<br>**#1**: *"fibromyalgia"* OR *"central sensitization"* OR *"chronic pain"*<br>**#2**: *"medical cannabis"* OR *"cannabis"* OR *"cannabinoids"*<br>**#3**: *"randomized controlled trial"* OR *"controlled clinical trial"* OR "randomized" OR "placebo" OR "randomly" OR "trial"<br>**#4**: #1 AND #2 AND #3	39
