# Supplementary material for: Cannabis for the Treatment of Fibromyalgia: A Systematic Review
Source: Biomedicines. 2023 Jun 2;11(6):1621. doi: 10.3390/biomedicines11061621 (PMC10295750; doi:10.3390/biomedicines11061621)
Supplement: Supplementary file 1 [file biomedicines-11-01621-s001.zip › Table S2.pdf]

Table S2. Excluded articles

Authors	Reason for exclusion
Lynch and Campbell (2011)	Review article
Habib and Avisar (2018)	Wrong study design
Berger et al. (2020)	Review article
Mazza (2021)	Wrong study design
Fitzcharles et al. (2021)	Review article
Habib and Aamar (2022)	Multiple pain etiologies
Boehnke et al. (2022)	Wrong study design
Van Dam et al. (2023)	Active clinical trial

References:

1. Habib G, Avisar I. The Consumption of Cannabis by Fibromyalgia Patients in Israel. *Pain Res Treat*. 2018;2018:7829427. Published 2018 Jul 22. doi:10.1155/2018/7829427
2. Mazza M. Medical cannabis for the treatment of fibromyalgia syndrome: a retrospective, open-label case series. *J Cannabis Res*. 2021;3(1):4. Published 2021 Feb 17. doi:10.1186/s42238-021-00060-6
3. Fitzcharles MA, Petzke F, Tölle TR, Häuser W. Cannabis-Based Medicines and Medical Cannabis in the Treatment of Nociplastic Pain. *Drugs*. 2021;81(18):2103-2116. doi:10.1007/s40265-021-01602-1
4. Habib G, Aamar S. Metabolic effects of medical cannabis treatment. *J Investig Med*. 2022;70(2):446-448. doi:10.1136/jim-2021-002059
5. van Dam CJ, van Velzen M, Kramers C, et al. Cannabis-opioid interaction in the treatment of fibromyalgia pain: an open-label, proof of concept study with randomization between treatment groups: cannabis, oxycodone or cannabis/oxycodone combination-the SPIRAL study. *Trials*. 2023;24(1):64. Published 2023 Jan 27. doi:10.1186/s13063-023-07078-6
6. Boehnke KF, Gagnier JJ, Matallana L, Williams DA. Cannabidiol Product Dosing and Decision-Making in a National Survey of Individuals with Fibromyalgia. *J Pain*. 2022;23(1):45-54. doi:10.1016/j.jpain.2021.06.007
7. Lynch ME, Campbell F. Cannabinoids for treatment of chronic non-cancer pain; a systematic review of randomized trials. *Br J Clin Pharmacol*. 2011;72(5):735-744. doi:10.1111/j.1365-2125.2011.03970.x
8. Berger AA, Keefe J, Winnick A, et al. Cannabis and cannabidiol (CBD) for the treatment of fibromyalgia. *Best Pract Res Clin Anaesthesiol*. 2020;34(3):617-631. doi:10.1016/j.bpa.2020.08.010
